# Long-Acting Injectable Antipsychotic Use in Patients with Schizophrenia and Criminal Justice System Encounters

**DOI:** 10.36469/jheor.2021.22979

**Published:** 2021-05-19

**Authors:** Madhav P. Bhatta, Saroj Bista, Antoine C. El Khoury, Eric G. Hutzell, Neeta Tandon, Douglas Smith

**Affiliations:** 1 Kent State University College of Public Health, Kent, OH; 2 Janssen Scientific Affairs LLC, Titusville, NJ; 3 County of Summit Alcohol, Drug Addiction, and Mental Health Services Board, Akron, OH

**Keywords:** arrest, incarceration, schizoaffective disorder, schizophrenia, criminal justice system, long-acting injectable, paliperidone palmitate

## Abstract

**Background:** Nonadherence to medication is prevalent in persons diagnosed with schizophrenia, thus increasing the likelihood of relapse, poor health outcomes, hospitalization, high treatment costs, and high rates of both violent and non-violent offenses.

**Objective:** To assess the association between long-acting injectable (LAI) antipsychotic use and criminal justice system encounters in patients with schizophrenia or schizoaffective disorder.

**Methods:** This retrospective follow-up study was conducted among patients aged ≥18 years treated for schizophrenia or schizoaffective disorder at a community mental health center in Akron, Ohio, between January 1, 2010, and June 15, 2016. The incidence of criminal justice system encounters at 6 months, 1 year, and 2 years pre- versus post-LAI antipsychotic initiation was assessed. A subanalysis was conducted for individuals with a history of prior arrest.

**Results:** Overall, the risk ratio (RR) of having an encounter with the criminal justice system was significantly lower for patients treated with LAI antipsychotics 1 year after initiation of treatment compared with a similar time period prior to initiation (RR [95% confidence interval (CI)]: 0.74 [0.59–0.93]; *P*<0.01) and 2 years (0.74 [0.62–0.88]; *P*<0.0001). Statistically significant reductions in criminal justice system encounters after treatment than before treatment were observed in the once-monthly paliperidone palmitate (PP1M) cohort. The incidence of arrests was lower in the 6-month (27 vs 85 arrests), 1-year (46 vs 132 arrests) and 2-year (88 vs 196 arrests) periods post-index LAI medication than in the corresponding periods pre-index LAI medication among individuals with a history of prior arrest.

**Conclusions:** Patients with schizophrenia or schizoaffective disorder who were initiated on a LAI antipsychotic medication, specifically PP1M, were less likely to have an encounter with the criminal justice system compared with a similar time period before the initiation of LAI treatment.

## INTRODUCTION

People with mental health disorders have a high incidence of poverty, unemployment, crime, victimization, family breakdown, homelessness, substance use, physical health problems, and stigma.^1^ Schizophrenia is a complex, chronic mental health disorder.^2^ Although it has a low lifetime prevalence of 0.7%, schizophrenia has received significant attention because people with this disorder often experience disability and serious physical, social, and economic consequences due to the severity of symptoms and the chronic nature of the disorder.^3,4^

Access to and consistent use of antipsychotic medications is an integral component of the clinical management of schizophrenia.^4^ Discontinuation is one of the main reasons for the lack of effectiveness of antipsychotic medication.^5^ Patients with schizophrenia often have poor adherence to antipsychotic medication, with up to 50% either partially adherent or non- adherent within 1 year after discharge from the hospital setting.^6^ Poor adherence to antipsychotic medication increases the likelihood of relapse, leading to poor health outcomes, a high risk of hospitalization, high treatment costs,^7,8^ and high rates of both violent and non-violent offenses.^9^ Conversely, high rates of adherence in individuals with mental illness are associated with a low risk of incarceration and low incarceration-related costs.^10^ In recent years, there has been an increased emphasis on strategies such as simplification of medication regimens, monotherapy, and the use of long-acting injectable (LAI) therapy to improve adherence, which may result in improved outcomes.^11,12^

Clinical practice guidelines highlight the potential benefit of long-acting injectable antipsychotic medications in patients who experience recurrent relapses related to partial or full non-adherence.^4,13^ Real-world studies suggest that patients with schizophrenia treated with LAI antipsychotic medication have higher rates of adherence and a reduced risk of hospitalization compared with patients treated with oral antipsychotics.^14,15^ Encounters with the criminal justice system are not uncommon in individuals with schizophrenia and can exacerbate prevailing social marginalization and disrupt access to treatment and to mental health services.^1^ Individuals with schizophrenia are overrepresented in the US prison population, with the prevalence estimated to range from 2.0% to 6.5% compared with 0.25% to 0.64% in the general population.^1,3^ The long-term goal of care for this patient group is to reduce the rate of incarceration and transfer patients to community mental health centers.^16^

A few studies have evaluated the impact that LAI antipsychotics may have among patients with schizophrenia in terms of risk of encounters with the criminal justice system.^17,18^ The Paliperidone Palmitate Research in Demonstrating Effectiveness (PRIDE) study—a prospective, open-label, randomized, 15-month study aiming to demonstrate the superiority of LAI antipsychotics over daily oral antipsychotics in delaying time to treatment failure including arrest and incarceration—found that the once-monthly paliperidone palmitate (PP1M) treatment delayed the time to first arrest by 49% compared with oral antipsychotics in patients with schizophrenia and a prior history of a criminal justice system encounter.^17^ In a subanalysis of the PRIDE study data, patients with chronic schizophrenia on oral medications were 37% more likely to be arrested compared with those on PP1M treatment.^18^ The PRIDE study was the first study to delineate the beneficial effect of LAIs in reducing the risk of a criminal justice system encounter in patients with schizophrenia. However, the PRIDE study was a clinical trial with willing participants and a high adherence rate; thus, its results may have limited generalizability to the general schizophrenia population. Furthermore, the PRIDE study included only one LAI (PP1M) with oral antipsychotic medication as the comparison group and had a short (15-month) follow-up period. A study examining the effect of LAI on the risk of a criminal justice system encounter in patients with schizophrenia receiving care at a community-based care provider would provide information on the real-world impact of LAI on risk reduction.

The present analysis assessed the association between LAI antipsychotic medication use and encounters with the criminal justice system among individuals receiving care for schizophrenia or schizoaffective disorder at a community mental health center. This study aims to provide further evidence on the benefits of continued antipsychotic medication treatment in mitigating societal burden including criminal justice system encounters.

## METHODS

### Study Design

This was a retrospective follow-up study using clinical data derived from a community-based service provider for individuals with mental illnesses in Summit County, Ohio (Community Support Services [CSS]), service claims data from the Summit County Alcohol, Drug Addiction, and Mental Health Services Board, and criminal justice data from the Summit County Jail records. Data from individuals aged ≥18 years who received treatment for schizophrenia or schizoaffective disorder from CSS between January 1, 2010, and June 15, 2016, were extracted from the three sources described above and were included in the analysis. Records of encounters with the criminal justice system were derived from the Summit County Jail through July 18, 2018. Only individuals receiving treatment with an LAI antipsychotic were included in the analysis. Everyone in the sample analyzed had at least 2 years of follow-up from the initiation of LAI treatment to the end of the study period. This study was approved by the Kent State University Institutional Review Board.

### Assessments

The primary exposure of interest was the start of treatment with one of the following LAI antipsychotic drugs (index LAI): aripiprazole, fluphenazine decanoate, haloperidol decanoate, PP1M, or risperidone. The primary endpoint was an encounter with the criminal justice system, as recorded within court records from the Summit County Jail. An encounter with the criminal justice system was defined as any time that a person was booked for a misdemeanor or felony offense. The outcomes assessed in this intent-to-treat analysis were the incidence of criminal justice system encounters at 6 months, 1 year, and 2 years pre- versus post-LAI initiation.

We compared the incidence of criminal justice system encounters between the pre- and post-LAI initiation periods (6 months, 1 year, and 2 years) overall and by the three drug categories (PP1M, first-generation antipsychotics [FGA], and other second-generation antipsychotics [SGA]). We also compared the risk of criminal justice system encounters among the three drug categories during the 2 years following LAI initiation. For the comparative analysis, individuals initiated on fluphenazine decanoate or haloperidol decanoate were combined into a single group (FGA); those on aripiprazole or risperidone were combined into a second group (other SGA); and those on PP1M formed a third group (PP1M only). Potential confounding variables included in the analysis were age, sex, race, substance use disorder, and prior history of arrest.

We performed a subanalysis (stratified analysis) of the risk of criminal justice system encounters for individuals with a history of prior arrest and/or substance use disorder. Substance use disorder diagnosis was based on criteria specified in the fourth edition of the Diagnostic and Statistical Manual of Mental Disorders (DSM-IV) and categorized as “yes” or “no.” Prior history of arrest was defined as the number of encounters with the criminal justice system within 5 years prior to starting the index medication. For the present analysis, history of arrest was categorized as “none” versus “one or more” arrests.

### Statistical Analysis

The baseline demographics and clinical characteristics of the study sample were summarized using descriptive statistics. Chi-square test and *t* test were used to compare proportions and means between males and females. The mean number of arrests and the incidence (proportion) of criminal justice system encounters during the three post-LAI initiation periods (6 months, 1 year, and 2 years) overall and stratified by a history of arrest and/or substance use disorder were also computed. Significant differences between pre- and post-index LAI data were identified using a 2-sided *P* value of <.05 (McNemar’s test).

The risk of criminal justice system encounters during the 2-year follow up period post-initiation was compared for the three LAI groups using the PROC GENMOD procedure for modified multivariable Poisson regression in SAS^®^. The confounding variables adjusted in the model included age, sex, race, substance use disorder, and prior history of arrest. The resulting risk ratios (RR) and associated 95% confidence intervals (CIs) are reported with FGA as the reference.

For the pre- and post-LAI initiation criminal justice system encounter analysis, matched-cohort analyses were performed to compare the incidence of at least one criminal justice system encounter before and after the initiation of an LAI index medication. The pre- and post-analysis of data from individual patients has some inherent benefits including the ability to match on fixed individual characteristics including sex, race, prior history of arrest, and substance use disorder since each patient serves as their own “control.” In that sense, there is a control group, although it is an internal control group rather than an external control group. Since the pre-and post-follow-up period was short (<2 years), age was essentially matched as well. RRs were computed to compare the risk of criminal justice system encounters overall and by the 3 LAI groups at 6 months, 1 year, and 2 years before and after the initiation of a medication using PROC GENMOD procedure for modified Poisson regression in SAS^®^. The RRs and associated 95% CIs and selected *P* values are reported. All analyses were conducted using SAS^®^, version 9.4, MS SQL Server 2017, and Tableau 2018.

## RESULTS

### Baseline Demographic and Clinical Characteristics

The analysis included 978 patients receiving treatment for schizophrenia or schizoaffective disorder from CSS between 2010 and 2016 (Table 1). Overall, 61.5% of patients were male and most of the patients were white (53.4%). The mean (standard deviation [SD]) age was 43.8 (13.5) years. Females were significantly older than males (47.3 years vs 41.6 years; *P*<0.0001). PP1M was the index LAI medication for more than half of the patients (58.9%; Table 1). Overall, 38.5% of patients had a substance use disorder as defined in the DSM-IV and 31.5% of patients had had at least one encounter with the criminal justice system during the 5 years prior to the start of the index LAI medication.

**Table 1.** Demographics and Baseline Clinical Characteristics of a Cohort of Individuals Receiving Care For Schizophrenia or Schizoaffective Disorder at a Community Mental Health Center

**Characteristics**	**Overall**	**Male**	**Female**	***P* Value**
	N (%)	n (%)	n (%)	
	978 (100)	602 (61.5)	376 (38.5)	
**Age, Years**				<0.0001
<30	191 (19.5)	147 (24.4)	44 (11.7)	
30 to <40	201 (20.6)	135 (22.4)	66 (17.6)	
40 to <50	222 (22.7)	124 (20.6)	98 (26.1)	
50 to <60	248 (25.4)	152 (25.3)	96 (25.5)	
≥60	116 (11.9)	44 (7.1)	72 (19.2)	
**Race**				0.2035
Black	433 (44.3)	260 (43.2)	173 (46.0)	
White	522 (53.4)	324 (53.8)	198 (52.7)	
Other	23 (2.4)	18 (3.0)	5 (1.3)	
**Current Substance Abuse**				<0.0001
Yes	376 (38.5)	272 (45.2)	104 (27.7)	
No	602 (61.5)	330 (54.8)	272 (72.3)	
**Prior Arrest**				<0.0033
0	670 (68.5)	385 (64.0)	285 (75.8)	
≥1	308 (31.5)	217 (36.0)	91 (24.2)	
**Long-acting Injectable Index Medication**				0.9284
Aripiprazole	62 (6.3)	37 (6.2)	25 (6.7)	
Fluphenazine Decanoate	77 (7.9)	50 (8.3)	27 (7.2)	
Haloperidol Decanoate	93 (9.5)	58 (9.6)	35 (9.3)	
Olanzapine	1 (0.1)	1 (0.2)	0 (0.0)	
Paliperidone Palmitate (Once-Monthly)	576 (58.9)	350 (58.1)	226 (60.1)	
Risperidone	169 (17.3)	106 (17.6)	63 (16.8)	

### Encounters with the Criminal Justice System

**Figure 1A-C.**
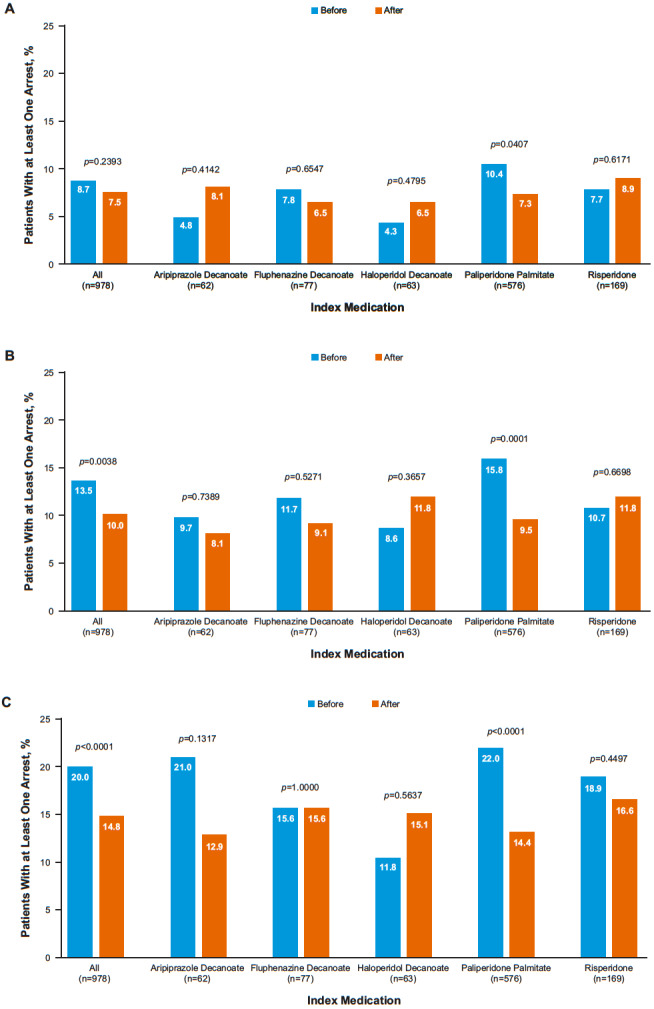
Proportion of Patients With at Least One Arrest A) 6-Months; B) 1-Year; and C) 2-Years Before and After the Start of the Index Medication

The incidence of a criminal justice system encounter (the proportion with at least one criminal justice system encounter) according to overall and index medications during the 6-month, 1-year, and 2-year periods before and after initiating an index LAI antipsychotic medication is shown in Figure 1A-C. Overall, of the 978 individuals in the study, 73, 98, and 145 had at least one arrest during the 6-month, 1-year, and 2-year follow-up periods, respectively. The overall incidence of arrest during the three time periods declined from 8.7, 13.5, and 20.0 to 7.5, 10.0, and 14.8 per 100 persons, before and after the LAI initiation, respectively. The incidence of arrest before the LAI initiation was the highest among those initiated on PP1M during the three periods.

During the 2-year follow-up period, those on PP1M were 33% less likely to have a criminal justice system encounter compared with those on the first-generation LAIs (fluphenazine and haloperidol) after adjustment for the potential confounding effects of age, sex, race, substance abuse disorder, and a history of prior arrest (RR=0.67; 95% CI: 0.47-0.96; *P*=0.0272). The difference observed in incidence of a criminal justice system encounter between those on other SGAs (aripiprazole and risperidone) compared with those on the first-generation LAIs was not statistically significant (RR=0.78; 0.52-1.18; *P*=0.3392).

The mean number (SD) and the risk of arrests before and after an index LAI antipsychotic medication initiation stratified by the previous history of arrest and/or substance use disorder is shown in Table 2. Among individuals with substance use disorder and an arrest during the 6-month, 1-year, and 2-year pre-index medication period, 26/69, 40/100, and 72/135, respectively, were arrested during the corresponding post-index LAI medication period. Thus, the proportion of patients with a prior history of arrest and substance use disorder who had no arrests in the post-index period was 62.3% in the 6-month, 60.0% in the 1-year, and 46.7% in the 2-year follow-up periods. This represents a significant reduction in the risk of arrest during the post-LAI medication period compared with the corresponding pre-LAI medication period (Table 2). The proportion of patients without a history of arrest and without a history of substance use disorder who remained without arrest for up to 2 years after starting LAI antipsychotic medication was 94.1%.

**Table 2.** Probability of Pre- and Post-LAI Antipsychotic Initiation Arrests in Persons with Schizophrenia or Schizoaffective Disorder With or Without a Prior History of Arrest and/or Substance Use Disorder

	**Before LAI Index Medication**		**After LAI Index Medication**		
	**Total No. of Patients**	**Mean (SD) Arrests per Patient**	**No. (%) of Patients With ≥1 Arrest**	**Mean (SD) Arrests per Patient**	**No (%) of Patients Without an Arrest**
**With a History of Arrest and SUD**					
6-month F/U	69	1.5 (0.8)	26 (37.7)*	0.6 (0.9)**	43 (62.3)
1-year F/U	100	1.8 (1.4)	40 (40.0)*	0.8 (1.3)**	60 (60.0)
2-year F/U	135	2.5 (2.1)	72 (53.3)*	1.3 (1.8)**	63 (46.7)
**With a History of Arrest Only**					
6-month F/U	16	1.3 (0.8)	1 (6.3)^†^	0.06 (0.3)**	15 (93.6)
1-year F/U	32	1.3 (0.7)	6 (18.8)*	0.3 (0.7)**	26 (81.3)
2-year F/U	61	1.9 (1.4)	16 (26.2)*	0.5 (0.9)**	45 (73.8)
**With a History of SUD Only**					
6-month F/U	307	-	23 (7.5)	0.1 (0.3)**	284 (92.5)
1-year F/U	276	-	26 (9.4)	0.1 (0.5)**	250 (90.6)
2-year F/U	241	-	25 (10.4)	0.2 (0.7)**	216 (89.6)
**Without a History of Arrest and Without a History of SUD**					
6﻿-﻿month F/U	586	-	23 (3.9)	0.05 (0.3)**	563 (96.1)
1-year F/U	570	-	26 (4.6)	0.07 (0.4)**	544 (95.4)
2-year F/U	541	-	32 (5.9)	0.08 (0.4)**	509 (94.1)

Abbreviations: F/U, follow-up; LAI, long-acting injectable; SD, standard deviation; SUD, substance use disorder.

*P<0.0001 and *^†^P*=0.0348 for the decline in the proportion with arrest post-LAI initiation compared to the baseline of 100% with an arrest.

**P<0.0001 for the difference in the mean number of arrests post-LAI initiation compared to pre-LAI initiation.

Comparisons of the risk of a criminal justice system encounter before versus after initiating an index LAI antipsychotic medication are shown in Table 3. The overall risk of a criminal justice system encounter was significantly lower for patients at 1 year and 2 years after initiation of LAI antipsychotic medication compared with 1 year and 2 years before LAI initiation (RR=0.74, 95% CI=0.59-0.93 at 1 year post-LAI initiation; and RR=0.74, 95% CI=0.62-0.88 at 2 years post-LAI initiation). Moreover, individuals receiving treatment with PP1M had a significant reduction in risk of a criminal justice system encounter after treatment initiation compared with before treatment initiation (RR=0.60, 95% CI=0.46-0.80 at 1 year post-PP1M initiation; and RR=0.65; 95% CI=0.53-0.81 at 2 years post-PP1M initiation).

**Table 3.** Comparison of the Risk of Encounters with the Criminal Justice System After Initiating Index LAI Antipsychotic Treatment in a Cohort of Persons With Schizophrenia or Schizoaffective Disorder

**≥1 Encounter With the Criminal Justice System Pre-index Medication Initiation**	**≥1 Encounter with the Criminal Justice System Post-index Medication Start Initiation**					
	**Within 6 Months**		**Within 1 Year**		**Within 2 Years**	
	**Yes**	**No**	**Yes**	**No**	**Yes**	**No**
Overall (N=978)						
Yes	27	58	46	86	88	108
No	46	847	52	794	57	725
^a^RR (95% CI)	0.86 (0.65-1.14)		0.74 (0.59-0.93)*		0.74 (0.62-0.88)***	
Paliperidone Palmitate Once-Monthly (n=576)						
Yes	16	43	30	61	54	73
No	26	491	25	460	29	420
^a^RR (95% CI)	0.71 (0.50-1.05)		0.60 (0.46-0.80)**		0.65 (0.53-0.81)***	
Other Second-Generation Antipsychotic Medications (n=231)	Aripiprazole (n=62) + Risperidone (n=169)					
Yes	7	9	9	15	21	24
No	13	202	16	191	15	171
^a^RR (95% CI)	1.25 (0.70-2.23)		1.04 (0.63-1.72)		0.80 (0.56-1.14)	
First-Generation Antipsychotic Medications (n*=*170)	Haloperidol Decanoate (n=93) + Fluphenazine Decanoate (n=77)					
Yes	4	6	7	10	13	11
No	7	153	11	142	13	133
^a^RR (95% CI)	1.10 (0.51-2.38)		1.05 (0.59-1.90)		1.08 (0.69-1.70)	

Abbreviations: CI, confidence interval; LAI, long-acting injectable; RR, risk ratio.

*P<0.01; **P=0.001; ***P<0.0001.

^a^Matched RR (95% CI) comparing effect of LAI antipsychotic treatment on the risk of a criminal justice system encounter after initiating index LAI antipsychotic drug treatment compared to the same length of time period before the initiation of LAI. Matched RR was computed using PROC GENMOD procedure for Modified Poisson regression methods in SAS®.

## DISCUSSION

The present retrospective follow-up analysis showed that treatment of patients with schizophrenia or schizoaffective disorder with LAI antipsychotics was associated with a lower risk of encounters with the criminal justice system compared with the same period before the initiation of LAI treatment. The reduction in the incidence of criminal justice system encounters after LAI initiation compared with pre-LAI initiation continued to increase with the increasing follow-up period. During the 2-year post-LAI initiation period, the incidence was 14.8% compared with 20.0% during the pre-LAI initiation 2-year period. Those on PP1M were significantly less likely to be arrested during the 2-year follow-up period after the LAI initiation compared with those on the first-generation LAIs; no such difference was observed between the other second-generation and first-generation LAIs. Thus, the overall decline in the risk of arrest seen during the post-LAI initiation periods was primarily driven by the decline in risk among those treated with PP1M. In fact, the greatest decline in the risk of arrest was observed in patients treated with PP1M, for as long as 1 year and 2 years after starting treatment compared with 1 year and 2 years before starting treatment. The pre-LAI initiation incidence of arrest was highest among those who were initiated on PP1M, suggesting the potential for confounding factors including a history of prior arrest. Although the post-LAI decline resulted in a similar risk of arrest in the PP1M group compared with the other two groups, following adjustment for age, race, substance use disorder, and history of prior arrest, the post-LAI risk was significantly (33%) lower among those on PP1M compared with those on the first-generation LAIs.

The incidence of arrest among patients with a history of criminal justice system encounters decreased significantly after LAI antipsychotic therapy was initiated. The risk of arrest was higher in patients with a prior history of an encounter with the criminal justice system than in those without such a history, which is consistent with earlier reports of prior incarceration increasing the risk of subsequent incarceration for people with schizophrenia.^19,20^

Previously, the PRIDE study was conducted in patients with schizophrenia at high risk for treatment non-adherence, including those with involvement with the criminal justice system or with comorbid substance use.^17^ Findings from the PRIDE study, a randomized study designed to reflect the real world, demonstrated the superiority of PP1M over daily oral antipsychotics in delaying time to treatment failure. Results showed that arrest/incarceration and hospitalization rates were lower for patients treated with PP1M compared with those treated with oral antipsychotics (33.6% vs 45%, respectively).^17^ The present retrospective analysis supports findings from the PRIDE study, highlighting that outcomes in a vulnerable population such as patients with schizophrenia at risk for treatment failure may be impacted by antipsychotic treatment choice.

An earlier study found that crimes committed by patients with schizophrenia were predominantly non-violent in nature and that offenders were primarily male.^19^ Substance use, which is common among individuals with schizophrenia and may be a criminal offense, could account for the increased risk of an encounter with the criminal justice system observed in this study and others.^21^ The reduced risk of arrest for patients treated with PP1M in the present analysis suggests that PP1M treatment may reduce the risk of committing non-violent offenses associated with shorter periods of incarceration, such as those associated with drug possession, drug dealing, and/or property offenses. However, it is important to note that the underlying nature of offenses resulting in an encounter with the criminal justice system was not investigated in this study. Although schizophrenia is a disease with a low prevalence, it generates substantial health, societal, and economic burden, which impacts not only patients but also their families, caregivers, and the wider society. Many people affected by schizophrenia experience fear and embarrassment about the signs and symptoms of the disease, uncertainty about prognosis, a substantial care burden, stigma, and lack of social support.^22^ A recent systematic review of 24 countries estimated annual costs of schizophrenia to be in the range of US$94 million to US$102 billion (taking into account direct and indirect costs, as well as costs relating to deterioration in quality of life for patients, their families, and their friends), which translates to 0.02%-5.46% GDP.^23^ The cost of incarceration in the United States, including costs borne by correctional institutions, incarcerated persons, and their families, their children, and their communities, is estimated to be in the region of US$1 trillion.^24^ Furthermore, a recent analysis calculated the cost of schizophrenia to US law enforcement to be US$7 billion, nearly 5% of the total cost impact of the disease. Incarceration costs were estimated to be US$3.6 billion, and judicial, legal, and police protection costs were estimated to be US$3.5 billion.^25^ Interventions to reduce the risk of arrest among patients with schizophrenia, therefore, may provide substantial benefits to patients, their families, and society, including reductions in financial cost. As observed in this study, treatment with LAIs, especially PP1M, appears to reduce the risk of arrest, thus highlighting the potential benefits of LAIs.

### Limitations

This retrospective follow-up study has several limitations, including limited geographical coverage; the fact that retrospective studies of health-care databases carry inherent limitations as variables used in the analysis were not set a priori. Therefore, missing data, or data inconsistently or incorrectly recorded, and the absence of detailed patient information, including potential confounding factors such as history of antipsychotic medication use, may have influenced treatment selection and patient adherence data. Since the medication at the start of the follow-up was used in the analysis, we cannot be certain that an individual remained on the same medication during the entire follow-up period. However, individuals would have been on a treatment, if not the index medication, during the follow-up period. Therefore, the observed effect of medication between the groups is likely an underestimation of the actual effect. Future prospective cohort studies with more complete medication adherence data are recommended to strengthen the findings of the study.

Although this analysis was conducted on a large population overall, the low numbers across the other antipsychotic treatment groups, aside from PP1M, offer limited statistical power and reduced the ability to detect differing incidences between individual LAI treatment groups in encounters with the criminal justice system. Although we increased the statistical efficiency by combining similar groups of LAIs (first generation and second generation), such an approach also results in the loss of ability to examine the effects of individual drugs. Furthermore, we cannot be certain that patients resided in Summit County for the entire study period based on the source data; however, the percentage of patients who received services only in Summit County (97.8%) and the mean (SD) duration of service (5.06 [1.09] years between first and last service received from CSS) suggest a geographically stable population.

The major limitations of the pre/post design of this study should also be acknowledged. Since an individual served as their own control, time-independent characteristics such as sex and race are controlled through matching. However, time-dependent characteristics such as age and differences in services and programs offered through the center, in addition to the treatments themselves, may have also impacted the results. In the multivariable Poisson regression model, age did not have a significant interaction with time. We also have matched on the duration of the pre- and post-LAI initiation follow-up period, and the follow-up period is only 2-years. More than 97% (951/978) of the individuals in the study were receiving other services before the LAI initiation and all continued receiving them afterward. The fact that a significant effect in pre- and post- LAI initiation risk of a criminal justice system encounter was only observed in one LAI group suggests that the effects of other programs and services may be limited. Nevertheless, we cannot rule out the potential confounding effects of other unmeasured characteristics.

Managing patients with schizophrenia in the community, especially those with a history of incarceration, presents challenges. Many patients with schizophrenia are released from jail with little or no notice.^26^ Around one-third are released within 7 days of incarceration and approximately 50% in less than 1 month.^26^ Furthermore, <20% of incarcerated persons with schizophrenia served a sentence long enough to allow planning and implementation of mental health support upon release.^26^ It has been reported that provision of an adaptive service following release from incarceration is associated with lower recidivism in the first 90 days after release.^27^ LAI antipsychotics may have an important role in supporting patients with schizophrenia who are released from jail, especially for bridging any intervening time between release and the implementation of community mental health support.

## CONCLUSIONS

Individuals with schizophrenia or schizoaffective disorder who were initiated on an LAI antipsychotic medication, and specifically those started on PP1M, were less likely to have an encounter with the criminal justice system compared with the period before the initiation of LAI antipsychotic treatment. Among those with a prior encounter with the criminal justice system and substance use disorder, a significant reduction in the incidence of at least one arrest was observed during a 2-year follow-up period after LAI antipsychotic treatment initiation, specifically PP1M. These results highlight the potential effectiveness of LAI antipsychotic treatment in reducing the risk of arrest among those with a prior history of encounters with the criminal justice system.

### AUTHOR CONTRIBUTIONS

MPB: conceptualization, data curation, formal analysis, funding acquisition, investigation, methodology, project administration, resources, software, supervision, validation, visualization, and writing (original draft, review & editing) SB: conceptualization, data curation, formal analysis, investigation, methodology, project administration, software, validation, visualization, and writing (original draft, review & editing)

ACE-K: conceptualization, formal analysis, funding acquisition, methodology, project administration, resources, supervision, visualization, writing (review & editing)

EGH: conceptualization, data curation, formal analysis, funding acquisition, investigation, methodology, project administration, resources, software, supervision, validation, visualization, and writing (original draft, review & editing)

NT: conceptualization, formal analysis, funding acquisition, methodology, project administration, resources, supervision, visualization, and writing (review & editing)

DS: conceptualization, data curation, funding acquisition, project administration, resources, supervision, and writing (review & editing)

### ETHICAL DISCLOSURE

All the data management and analyses were conducted by ADM Board and Kent State University personnel. MPB and SB are employees of Kent State University. EGH and DS have no conflicts of interest. ACE-K and NT are employees of Janssen Pharmaceuticals.

### DATA SHARING STATEMENT

The data sharing policy of Janssen Pharmaceutical Companies of Johnson & Johnson is available at https://www.janssen.com/clinical-trials/transparency. As noted on this site, requests for access to the study data can be submitted through Yale Open Data Access (YODA) project site at http://yoda.yale.edu/.
